# Polymorphisms in the cytochrome *P*450 genes *CYP1A2, CYP1B1, CYP3A4, CYP3A5, CYP11A1, CYP17A1*, *CYP19A1 *and colorectal cancer risk

**DOI:** 10.1186/1471-2407-7-123

**Published:** 2007-07-05

**Authors:** Lara Bethke, Emily Webb, Gabrielle Sellick, Matthew Rudd, Stephen Penegar, Laura Withey, Mobshra Qureshi, Richard Houlston

**Affiliations:** 1 Section of Cancer Genetics, Institute of Cancer Research, Surrey, UK

## Abstract

**Background:**

Cytochrome *P*450 (CYP) enzymes have the potential to affect colorectal cancer (CRC) risk by determining the genotoxic impact of exogenous carcinogens and levels of sex hormones.

**Methods:**

To investigate if common variants of *CYP1A2, CYP1B1, CYP3A4, CYP3A5, CYP11A1, CYP17A1 *and *CYP19A1 *influence CRC risk we genotyped 2,575 CRC cases and 2,707 controls for 20 single nucleotide polymorphisms (SNPs) that have not previously been shown to have functional consequence within these genes.

**Results:**

There was a suggestion of increased risk, albeit insignificant after correction for multiple testing, of CRC for individuals homozygous for *CYP1B1 *rs162558 and heterozygous for *CYP1A2 *rs2069522 (odds ratio [OR] = 1.36, 95% confidence interval [CI]: 1.03–1.80 and OR = 1.34, 95% CI: 1.00–1.79 respectively).

**Conclusion:**

This study provides some support for polymorphic variation in *CYP1A2 *and *CYP1B1 *playing a role in CRC susceptibility.

## Background

Recent studies have provided strong evidence that exposure to carcinogens, such as polycyclic aromatic hydrocarbons, heterocyclic amines and others in the diet, influences the risk of developing colorectal cancer (CRC) [1-3]. In addition to diet, cigarette smoking is another source of carcinogenic exposure relevant to the large bowel, and increased risk of CRC associated with smoking has been documented in several studies (reviewed in [4]). The genotoxic impact of carcinogen exposure is heavily influenced by a complex array of metabolic pathways, which includes the cytochrome *P*450 (CYP) enzyme system. The CYP enzymes also participate in the metabolism of a number of endogenous compounds, such as sex hormones and fatty acids, which are increasingly recognized to be relevant to CRC development [5]. Because wide inter-individual variations in activity of many of the CYP enzymes have been related to the existence of genetic polymorphisms, there is an opportunity to look for inherited metabolic susceptibilities to CRC.

In the present study, we have investigated the relationship between single nucleotide polymorphisms (SNP) in *CYP1A2, CYP1B1, CYP3A4, CYP3A5, CYP11A1, CYP17A1 *and *CYP19A1 *and CRC risk in a large study of 2,575 cases and 2,707 controls. Our rationale for analysing SNPs mapping to these CYP genes is that *CYP3A4 *and *CYP3A5 *are highly expressed in colonic tissue (reviewed in [6]) and variants of *CYP1A2 *and *CYP1B1 *have previously been associated with CRC risk [7]. Additionally, *CYP11A1, CYP17A1 *and *CYP19A1*, which encode sex hormone metabolising CYP enzymes, have been linked to risk of other types of cancer [8,9] and hence represent credible candidates as CRC predisposition genes.

## Methods

### Patients and control subjects

Patients with histologically confirmed adenocarcinomas, ascertained through an ongoing initiative at the Institute of Cancer Research/Royal Marsden Hospital NHS Trust (RMHNHST) (1,474 males, 1,101 females; mean age at diagnosis 59 years; SD ± 10.1) were included in the study. Healthy individuals were recruited at centers throughout the UK as part of the National Cancer Research Network Trial (1999–2002), the Royal Marsden Hospital Trust/Institute of Cancer Research Family History and DNA Registry (1999–2004), or the National Study of Colorectal Cancer Genetics Trial (2004), all established within the United Kingdom. Controls (836 males, 1,871 females; mean age 59 years; SD ± 10.9) were the spouses or unrelated friends of patients with malignancies. None of the controls had a personal history of malignancy at time of ascertainment. All patients and controls were British Caucasians, and there were no obvious differences in the demography of the two groups in terms of place of residence within the UK. Blood samples were obtained with informed consent and ethical review board approval in accordance with the tenets of the Declaration of Helsinki. DNA was extracted from samples using conventional methodologies and quantified using PicoGreen (Invitrogen).

### SNP genotyping

Genotyping of samples was performed using customized Illumina (San Diego, CA) Sentrix Bead Arrays according to the manufacturer's protocols. Annotated flanking sequence information for each SNP was derived from the University of California Santa Cruz (UCSC) Human Genome Browser (Assembly hg17). SNP selection was in part restricted to those that are amenable to genotyping using the array-based platform used. DNA samples with GenCall scores < 0.25 at any locus were considered "no calls." It has been proposed that alleles linked to complex disease are likely to reflect modest changes in gene activity, therefore non-coding SNPs are as likely as coding SNPs to be associated with complex disease risk [10]. Hence our analysis was largely based on the genotyping non-coding polymorphisms in introns and within 2 kb of the mRNA of *CYP1A2, CYP1B1, CYP3A4, CYP3A5, CYP11A1, CYP17A1 *and *CYP19A1*. We did however make use of genotypes generated from a genome-wide association study of coding SNPs we have recently performed (two SNPs in *CYP1B1 *and one in *CYP3A4 *and *CYP3A5A*) to assist in haplotype construction. Table 1 provides details of all 20 SNPs analysed in this current report.

**Table 1:** *CYP *gene polymorphisms analysed

**Gene**	**SNP**	**Details**	**Chr**	**Coordinate**^1^	**Allele**	**A**^2^**B**^2^	**Top strand**
***CYP1A2***	rs2069522	within 2kb 5' of mRNA	15	72826286	A	G	CAGATGGATGGGGAATCCAATAGAG [A/G]AACAGAGCATGTTTGAAGGCCATGA
***CYP1B1***	rs1800440	coding N453S	2	38209790	A	G	TTCTTGGACAAGGAHGGCCTCATCA [A/G]CAAGGACCTGACCAGCAGAGTGATG
	rs1056836	coding L432V	2	38209854	C	G	AAGTTCTCCGGGTTAGGCCACTTCA [C/G]TGGGTCATGATTCACAGACCACTGG
	rs2617266	intronic	2	38214195	A	G	GGCTGGTGCCCATGCTGGGGACAGA [A/G]AGGAGAAGGCGTGACACTCAGGGGT
	rs2567206	within 2kb 5' of mRNA	2	38215182	A	G	CGCTTCATCACAGCCACCTCCAATC [A/G]AGGCCGCACGGTGTCCCCAGAACCA
	rs162558	within 2kb 5' of mRNA	2	38215730	A	G	ACTTCAACCCGATAAAGTTCGCCGG [A/G]GCGCGGAGATTCGCCTCCTCCTGCC
	rs49785885	within 2kb 5' of mRNA	2	38216383	C	G	TGAGCTCCACTCGCGCTGCGAATCT [C/G]GTGCTATTATTGACCTGTTTACAGA
***CYP3A4***	rs4986910	coding M445T	7	99003175	A	G	TTTCATGTTCATGAGAGCAAACCTC [A/G]TGCCAATGCAGTTTCTGGGTCCACT
	rs2242480	intronic	7	99006117	A	G	TTTTACCCAATAAGGTGAGTGGATG [A/G]TACATGGAGAAGGAGGGAGGAGGTG
	rs2687116	intronic	7	99010594	A	C	AATAAAGCAGTTATTTTTAAGAGAG [A/C]AAGATAAATAAAAGGAAATAGTAGT
	rs4986907	coding R162Q	7	99012078	A	G	GATGTGTTGGTGAGAAATCTGAGGC [A/G]GGAAGCAGAGACAGGCAAGCCTGTC
	rs12721636	5' UTR	7	99026417	A	C	GGAAAGCTCCATGCACATAGCCCAG [A/C]AAAGAGCAACACAGAGCTGAAAGGA
	rs2740574	within 2kb 5' of mRNA	7	99026747	A	G	GAGGACAGCCATAGAGACAAGGGCA [A/G]GAGAGAGGCGATTTAATAGATTTTA
	rs11773597	within 2kb 5' of mRNA	7	99027102	C	G	CTGTCATGGCTCTGGTCCCTACCAG [C/G]GTGCTGTACACACAGTGGGCAACTA
***CYP3A5***	rs15524	3' UTR	7	99083850	A	G	AGCTTTCTTGAAGACCAAAGTAGAA [A/G]TCCTTAGAATAACTCATTCTCCACT
	ss49785886	coding T398N	7	98894887	A	C	AAAGGGTCAATGGTGGTGATTCCAA [A/C]TTATGCTCTTCACCATGACCCAAAG
	rs776746	intronic	7	98915190	A	G	CTTTAAAGAGCTCTTTTGTCTTTCA [A/G]TATCTCTTCCCTGTTTGGACCACAT
***CYP11A1***	rs2073475	within 2kb 5' of mRNA	15	72448947	A	G	TGCCCCACAACACTGGGGGAGGTGC [A/G]GAGGCCTGAACGGAAGTTGGGGTGG
***CYP17A1***	rs743572	5' UTR	10	104587142	A	G	GGTGCCGGCAGGCAAGATAGACAGC [A/G]GTGGAGTAGAAGAGCTGTGGCAACT
***CYP19A1***	rs10046	intronic	15	49290278	A	G	CTACTGATGAGAAATGCTCCAGAGT [A/G]GGTACTGACCAGCCTTCTCTAGTGT

^1^Human genome build 35, ^2^Top strand allele, encoded according to the convention adopted by Illumina, Inc. for strand specific genotype annotation in the absence of a reference genome

### Statistical methods

The relationships between homozygote and heterozygote carrier status and risk of CRC and between number of putative risk alleles carried and risk of CRC were assessed by means of odds ratios (ORs) with 95% confidence intervals (CIs), calculated using logistic regression (adjusting for age and sex). Where it was not possible to calculate ORs and their CIs by asymptotic methods, an exact approach was implemented. A likelihood ratio test was performed to evaluate the impact of each SNP on case-control status. To test for population stratification, the distribution of genotypes in controls was tested for departures from Hardy-Weinberg equilibrium (HWE). To investigate epistatic interactions, each pair of SNPs was evaluated by fitting a saturated logistic regression model and the log likelihood ratio statistic for comparison with the main effects model computed. To assess the level of linkage disequilibrium (LD) between SNPs, we calculated the pair-wise LD measure *D*' between consecutive pairs of markers in the genes *CYP1B1*, *CYP3A4*, and *CYP3A5 *using the expectation-maximization algorithm to estimate two-locus haplotype frequencies. The program PHASE [11,12] which implements a Markov chain Monte Carlo method was used to generate haplotypes. Haplotype frequencies in cases and controls were compared by the chi-squared statistic. We report p-values both before and after adjustment for multiple testing, which was carried out by means of the Bonferroni correction. All computations were undertaken using the statistical software packages Stata (Stata Corp, TX, USA) or LogXact (Cytel Inc., Cambridge, MA, USA).

## Results

Genotypes were obtained for 2,561 of the 2,575 cases (99.5%) and 2,695 of the 2,707 controls (99.6%). SNP call rates per sample for each of the 5,256 DNA samples were > 99.8% in cases and > 99.5% in controls. CYP genotypes and allele frequencies in CRC cases and controls are detailed in Table 2. Genotypic frequencies of SNPs in controls were similar to those previously documented in Caucasians and none were found to violate HWE in controls. Table 2 also details the risk associated with each genotype as quantified by the raw OR and the OR adjusted for age and gender, and their associated 95% CIs. Two SNPs showed some evidence of an association with CRC risk: rs162558, which maps 5' to *CYP1B1*, and rs2069522, which maps 5' to *CYP1A2*. Homozygotes for rs162558 in *CYP1B1 *and heterozygotes for rs2069522 in *CYP1A2 *were associated with a mildly increased risk of CRC (OR = 1.36, 95% CI: 1.03–1.80 and OR = 1.34, 95% CI: 1.00–1.79 respectively), although neither of these findings were significant after correction for multiple testing (adjusted p = 1.0). In the case of rs162558 in *CYP1B1*, heterozygotes showed no evidence of increased risk. The logistic regression model based on alleles did not yield any further significant associations.

**Table 2:** *CYP *genotype frequencies in colorectal cancer cases and controls

**Gene**	**SNP**	**MAF (cases/controls)**	**Genotype**	**Cases**	**Controls**	**Raw OR (95% CI)**	**Adjusted OR (95% CI)**	***P***^2^
***CYP1A2***	rs2069522	0.022	AA	2444	2597	1.00 (ref)	1.00 (ref)	0.05
		0.017	**AB**	**115**	**94**	**1.30 (0.98–1.72)**	**1.34 (1.00–1.79)**	
			BB	0	0	--	--	
***CYP1B1***	rs1800440^1^	0.178	AA	1734	1790	1.00 (ref)	1.00 (ref)	0.15
		0.182	AB	739	828	0.92 (0.82–1.04)	0.90 (0.79,1.01)	
			BB	86	76	1.17 (0.85–1.60)	1.13 (0.81,1.57)	
	rs1056836^1^	0.452	AA	519	538	1.00 (0.86–1.17)	1.01 (0.86,1.19)	0.91
		0.453	AB	1277	1365	0.97 (0.86–1.10)	0.98 (0.86,1.12)	
			BB	763	792	1.00 (ref)	1.00 (ref)	
	rs2617266	0.279	AA	203	197	1.11 (0.90–1.36)	1.06 (0.85–1.33)	0.69
		0.271	AB	1022	1062	1.03 (0.92–1.16)	1.05 (0.93–1.18)	
			BB	1333	1430	1.00 (ref)	1.00 (ref)	
	rs2567206	0.280	AA	205	199	1.11 (0.90–1.36)	1.06 (0.85–1.33)	0.71
		0.271	AB	1019	1062	1.03 (0.92–1.15)	1.05 (0.93–1.18)	
			BB	1332	1429	1.00 (ref)	1.00 (ref)	
	rs162558	0.217	AA	1582	1668	1.00 (ref)	1.00 (ref)	0.10
		0.210	AB	847	918	0.97 (0.87–1.09)	1.02 (0.90–1.15)	
			**BB**	**132**	**108**	**1.29 (0.99–1.68)**	**1.36 (1.03–1.80)**	
	ss49785885	0	AA	2561	2691	1.00 (ref)	1.00 (ref)	0.50
		0.0004	AB	0	2	0.44 (0–5.60)	0.44 (0–5.60)	
			BB	0	0	--	--	
***CYP3A4***	rs4986910^1^	0.007	AA	2522	2646	1.00 (ref)	1.00 (ref)	0.56
		0.007	AB	38	40	1.00 (0.64–1.56)	1.15 (0.72,1.86)	
			BB	0	0	--	--	
	rs2242480	0.089	AA	24	26	0.95 (0.55–1.67)	0.94 (0.53–1.67)	0.36
		0.097	AB	410	471	0.90 (0.78–1.04)	0.89 (0.77–1.04)	
			BB	2127	2198	1.00 (ref)	1.00 (ref)	
	rs2687116	0.035	AA	2385	2505	1.00 (ref)	1.00 (ref)	0.98
		0.036	AB	173	187	0.97 (0.78–1.20)	0.98 (0.78–1.22)	
			BB	3	3	1.05 (0.21–5.21)	1.00 (0.19–5.26)	
	rs4986907^1^	0.0006	AA	0	0	--	--	0.28
		0.0002	AB	3	1	3.16 (0.33–30.37)	3.32 (0.32–34.09)	
			BB	2554	2688	1.00 (ref)	1.00 (ref)	
	rs12721636	0.0008	AA	0	0	--	--	0.19
		0.0004	AB	4	2	2.11 (0.39–11.51)	2.10 (0.30–23.29)	
			BB	2556	2691	1.00 (ref)	1.00 (ref)	
	rs2740574	0.034	AA	2389	2508	1.00 (ref)	1.00 (ref)	0.94
		0.035	AB	170	181	0.99 (0.79–1.22)	0.99 (0.78–1.24)	
			BB	2	3	0.70 (0.12–4.19)	0.73 (0.12–4.66)	
	rs11773597	0.066	AA	12	13	0.97 (0.44–2.13)	1.08 (0.48–2.43)	0.91
		0.067	AB	312	335	0.98 (0.83–1.15)	0.97 (0.81–1.15)	
			BB	2237	2347	1.00 (ref)	1.00 (ref)	
***CYP3A5***	rs15524	0.076	AA	2186	2261	1.00 (ref)	1.00 (ref)	0.24
		0.085	AB	360	408	0.91 (0.78–1.06)	0.92 (0.79–1.08)	
			BB	15	24	0.65 (0.34–1.24)	0.62 (0.31–1.21)	
	ss49785886^1^	0.004	AA	1	0	1.05 (0.03-∞)	1.05 (0.03-∞)	0.06
		0.007	AB	20	36	0.58 (0.34–1.01)	0.57 (0.32–1.02)	
			BB	2539	2655	1.00 (ref)	1.00 (ref)	
	rs776746	0.067	AA	12	16	0.78 (0.37–1.65)	0.75 (0.35–1.63)	0.56
		0.073	AB	320	363	0.92 (0.78–1.08)	0.93 (0.79–1.10)	
			BB	2225	2312	1.00 (ref)	1.00 (ref)	
***CYP11A1***	rs2073475	0.155	AA	64	62	1.11 (0.77–1.58)	1.09 (0.75–1.58)	0.43
		0.146	AB	667	661	1.08 (0.95–1.22)	1.09 (0.95–1.24)	
			BB	1829	1958	1.00 (ref)	1.00 (ref)	
***CYP17A1***	rs743572	0.379	AA	995	1045	1.00 (ref)	1.00 (ref)	0.86
		0.383	AB	1192	1234	1.01 (0.90–1.14)	1.00 (0.88–1.13)	
			BB	374	416	0.94 (0.80–1.11)	0.96 (0.80–1.14)	
***CYP19A1***	rs10046	0.472	AA	714	755	1.00 (ref)	1.00 (ref)	0.37
		0.467	AB	1272	1364	0.99 (0.87–1.12)	1.00 (0.88–1.15)	
			BB	572	575	1.05 (0.90–1.23)	1.11 (0.94–1.30)	

MA, minor allele; OR, odds ratio; CI, confidence interval; *p*, likelihood ratio test *p*-value; A, B, strand specific allele designations defined in Table 1; ^1^Hum Mol Genet. 2006 Nov 1;15(21):3263-71. ^2^unadjusted *p*-values, adjusted *p *= 1.0 (not shown)

Data on site of tumour and Duke's stage of tumour were available for 2,544 and 587 cases respectively. Of the patients for whom site of tumour was known,1,585 had colon cancer and 959 had rectal tumours. Of the patients for whom data regarding Duke's stage were available, 52 were stage A, 227 were stage B, 305 were stage C and 3 were stage D. Stratification of the analysis by each of these categories did not significantly affect findings.

Estimation of haplotypes in the genes with multiple SNPs resulted in 5, 4 and 3 common haplotypes (i.e. > 1%) for *CYP1B1, CYP3A4 *and *CYP3A5 *respectively. There was strong LD between the five common SNPs in *CYP1B1 *(*D*' > 0.97 for each pair of SNPs), the four common SNPs in *CYP3A4 *(*D*' > 0.95 for each pair of SNPs) and the three SNPs in *CYP3A5 *(*D*' > 0.84 for each pair of SNPs). When haplotype frequencies were compared in patients and controls no increased evidence of a relationship with CRC was apparent (Table 3). The joint effects of the polymorphisms on the risk of CRC were evaluated. Twelve pairs of the SNPs displayed nominal evidence of interaction at the 5% level, but none were significant after applying a Bonferroni correction for the 105 likelihood ratio tests undertaken.

**Table 3:** *CYP *haplotype frequencies in colorectal cancer cases and control

**Gene**	**Haplotype**^1^	**% frequency (standard error)**		***p***
		**Controls**	**Cases**	
***CYP1B1***	ABAAAA	26.85 (0.024)	27.7 (0.022)	0.49
	AABBAA	24.11 (0.038)	23.6 (0.037)	0.68
	AABBBA	20.91 (0.033)	21.5 (0.033)	0.63
	BBBBAA	18.01 (0.031)	17.6 (0.029)	0.73
	ABBBAA	9.58 (0.035)	8.98 (0.040)	0.46
***CYP3A4***	ABABBAB	82.64 (0.043)	83.59 (0.026)	0.36
	ABABBAA	6.70 (0.0071)	6.55 (0.016)	0.83
	AAABBAB	6.30 (0.043)	5.54 (0.028)	0.24
	AABBBBB	3.35 (0.0079)	3.32 (0.0086)	0.94
***CYP3A5***	ABB	90.83 (0.0056)	91.90 (0.0033)	0.17
	BBA	7.32 (0.0093)	6.66 (0.0027)	0.72
	BBB	1.14 (0.0095)	0.96 (0.0027)	0.24

^1^The order of genotypes is the order of SNPs as listed in Table 1, haplotypes with frequency less than 1% not shown.

## Discussion

Two of the variants we evaluated, *CYP1A2 *rs2069522 and *CYP1B1 *rs162558, showed mild evidence of an impact on CRC risk. For *CYP1B1 *rs162558, homozygosity was associated with increased CRC risk, and for *CYP1A2 *rs2069522 heterozygosity was associated with risk, although these associations were not significant after correction for multiple testing. The Bonferroni correction is based on the assumption that tests are independent. This is a conservative adjustment for these data due to the high levels of LD in *CYP1B1*, *CYP3A4 *and *CYP3A5*. However, the smallest observed unadjusted p-value of 0.05 would not be significant after correction for multiple testing even if a less conservative adjustment (such as adjusting for the ~7 independent tests once LD is taken into account) were used. Conflicting results from other studies have found these genes to be either associated or not associated with CRC risk [7,13]. Discrepancies between these previous studies may be due to small sample sizes, confounding interactions with environmental or genetic risk factors, and the choice of SNPs to be genotyped. We have tested 2575 cases and 2707 controls, over 5-fold more than any previous study of CRC involving these genes, and hence had far more power to detect moderate increases in risk.

We acknowledge that the choice of particular SNPs and/or interactions with other risk factors could moderate the observed results in any association study. Previous studies have implicated polymorphisms in the cytochrome *P*450 genes *CYP1A1*, *CYP2A6*, *CYP2C9*, and *CYP2C19 *in risk of CRC (reviewed in [14]). However the small sample sizes of many early association studies may have led to misleading results. While the precise nature of the role of the CYP genes in CRC risk is not yet clear, our findings expand the body of knowledge about these genes, and we hope they will contribute to the development of further research in this area.

As the genotypic frequencies of SNPs in our controls were similar to those previously documented in Caucasians and none were found to violate Hardy-Weinberg equilibrium there is no evidence that cryptic relatedness or population stratification impacted on study findings. Although we do not have participant rate data it seems unlikely that any selection from this would bias study findings (i.e. specific genotype more likely to have been ascertained).

SNP rs2069522 is located -2847 bp relative to *CYP1A2*, within a putative region of bi-directional suppressive transcriptional control (position -1329 to -4412) for both *CYP1A1 *and *CYP1A2 *genes [15], hence it is possible that this sequence change may influence transcription of CYP1A1 and CYP1A2 proteins. Previously, SNPs in both *CYP1A1 *and *CYP1A2 *have been associated with colorectal adenomas and carcinomas [7,16], therefore the observation of some relationship between *CYP1A2 *rs2069522 and risk in our study is not without precedent. Moreover, *CYP1A2 *is critical for the metabolic activation of dietary heterocyclic aromatic amines (HCA) which are mutagenic and have been implicated in the development of CRC [17-23].

SNP rs162558 is located -1548 bp relative to *CYP1B1*. *CYP1B1 *activates several human procarcinogens by an aryl hydrocarbon receptor-aryl hydrocarbon receptor nuclear translocator pathway [24,25]. *CYP1B1 *is overexpressed in colorectal adenocarcinomas relative to normal colon tissue [26], and a variant with increased activity towards several substrates including sex hormones has been associated with increased risk of CRC [7]. Our data support the evidence that genetic variants in *CYP1B1 *may be associated with CRC risk.

*CYP19A1 *rs10046 has previously been reported to influence levels of estradiol [8] and possibility impact on breast cancer risk. While estrogens undoubtedly affect CRC risk, in our study we found no evidence that variation in *CYP19A1 *defined by rs10046 had an impact on CRC risk.

Our study is large compared to contemporaneous studies, and although for the majority of SNPs assayed our analysis has 80% power to detect a relative risk of 1.5, inevitably we cannot entirely exclude a small effect in risk of CRC associated with the variants analysed. Moreover, as there is increasing evidence of a gene-environment effect with respect to CRC risk some of the polymorphisms may mediate CRC risk in the context of specific dietary risk factors. The primary aim of our study design was to acquire enough samples to achieve statistically significant results for the identification of common, low penetrance alleles in CRC. The addition of gene-environment interaction to our aims would require a vastly larger number of samples in order achieve significance. We hope that as technological advances enable studies of this nature to include larger numbers of patient samples, reliable information regarding carcinogen exposure and diet will be informative, but the collection and analysis of environmental factors was unfortunately not possible on the scale of our study.

## Conclusion

This study provides some support for polymorphic variation in *CYP1A2 *and *CYP1B1 *playing a role in CRC susceptibility.

## Competing interests

The author(s) declare that they have no competing interests.

## Authors' contributions

LB analysed the data and drafted the manuscript. EW performed the statistical analysis and helped to draft the manuscript. GS and MR participated in the design and coordination of the study. SP, LW, and MQ carried out the study. RH conceived of the study, participated in its design and coordination, and helped to draft the manuscript.

## Pre-publication history

The pre-publication history for this paper can be accessed here:


